# Supplementary material for: Electrochemical Sensor Based on Laser-Induced Graphene for Carbendazim Detection in Water
Source: Foods. 2023 Jun 6;12(12):2277. doi: 10.3390/foods12122277 (PMC10297698; doi:10.3390/foods12122277)

Supporting Information

Electrochemical Sensor Based on Laser-induced Graphene for Carbendazim Detection in Water

Li Wang, Mengyue Li, Bo Li, Min Wang, Hua Zhao and Fengnian Zhao *

College of Chemistry and Materials Engineering, Beijing Technology and Business University
*Correspondence: zhaofn@btbu.edu.cn

Figure S1. Characterization of the prepared LIG electrode. DPV curves of LIG electrode prepared by optimizing (a) power and (b) scanning speed. (c) SEM image of porous LIG.

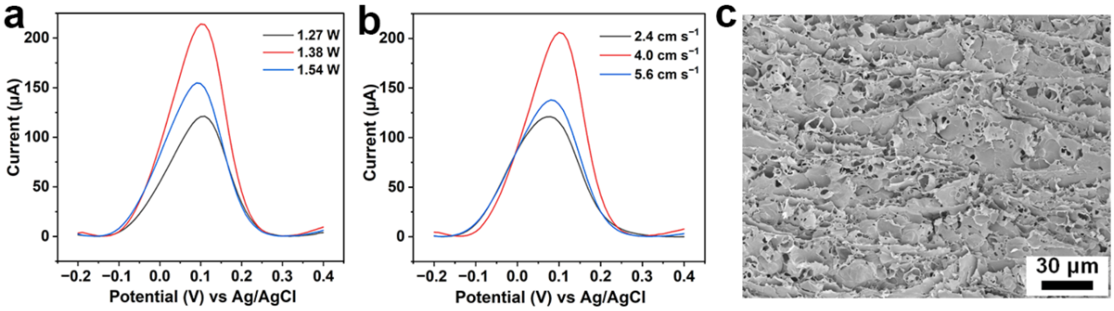

Figure S2. Images of the LIG-based three-electrode sensor. (a) The size parameters and (b) the image of the LIG electrode.

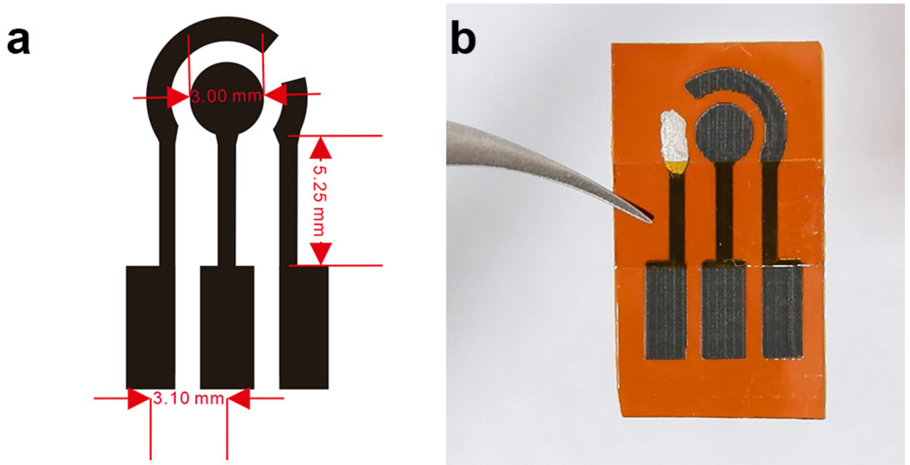

Figure S3. XPS fittings for C 1s of LIG.

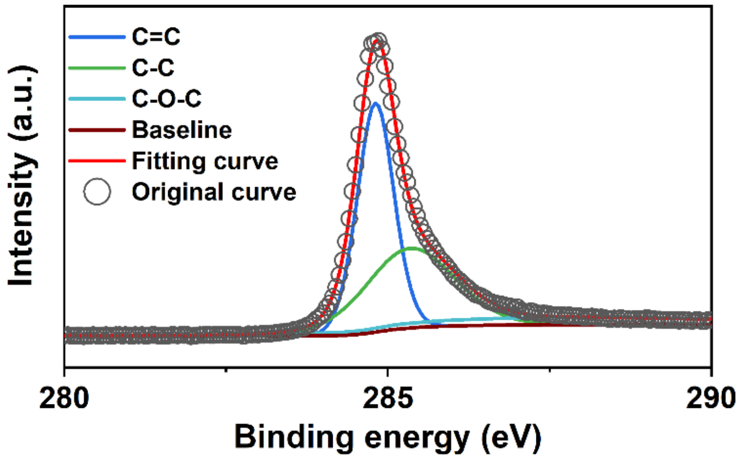

Figure S4. Characterization of the prepared LIG/Pt sensor. (a) DPV response of LIG/Pt sensors with different electrodeposition cycles (10, 20, and 30 are the CV cycles during the electrodeposition). (b) SEM image of the prepared LIG/Pt sensor.

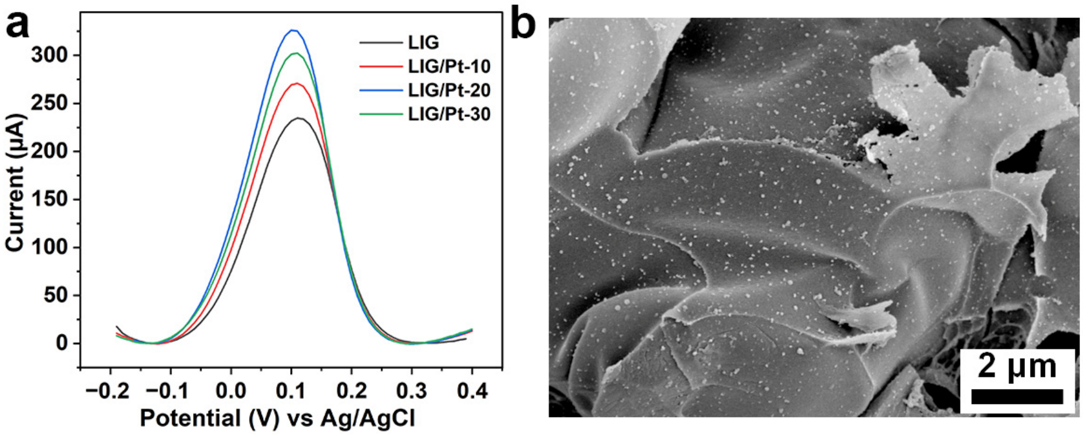

Figure S5. Measurement of (a) anti-interference performance, (b) selectivity, (c) reproducibility, and (d) stability of LIG/Pt sensor.

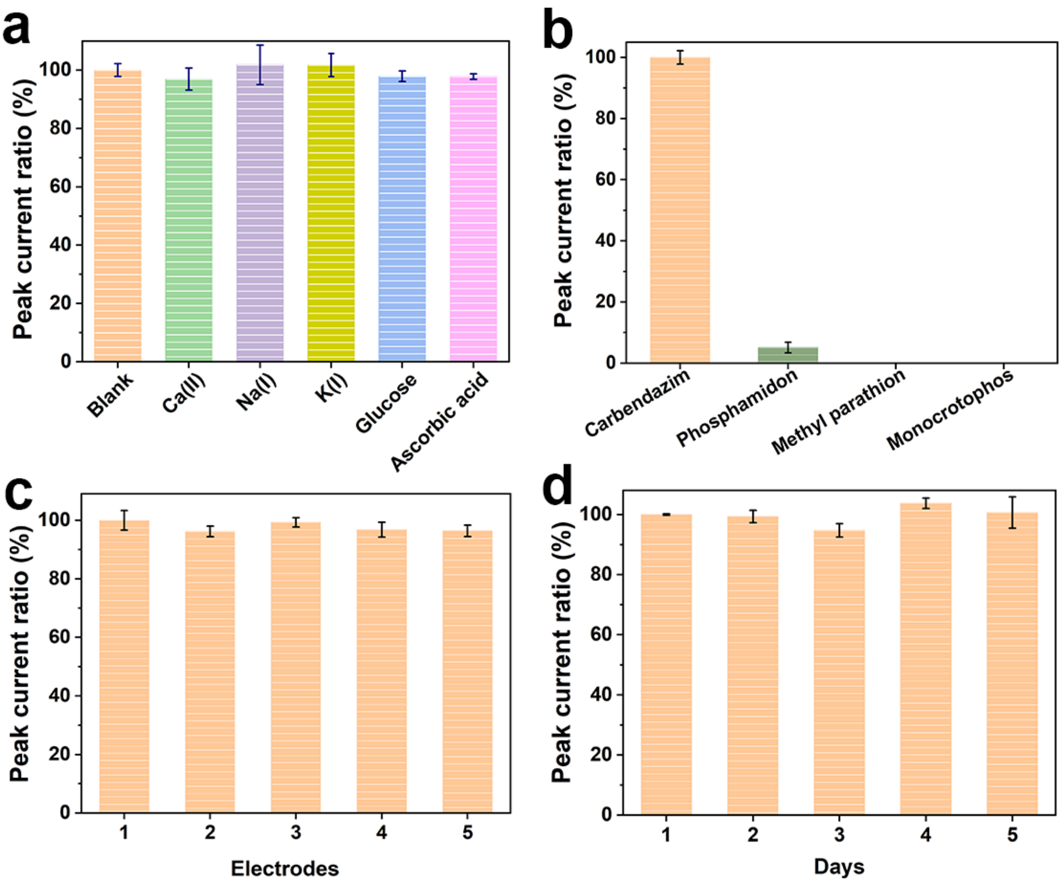

Supplement: Supplementary file 1 [file foods-12-02277-s001.zip › foods-2401234-supplementary.pdf]
